# Supplementary material for: Associating Increased Chemical Exposure to Hurricane Harvey in a Longitudinal Panel Using Silicone Wristbands
Source: Int J Environ Res Public Health. 2022 May 30;19(11):6670. doi: 10.3390/ijerph19116670 (PMC9180596; doi:10.3390/ijerph19116670)
Supplement: Supplementary file 1 [file ijerph-19-06670-s001.zip › ijerph-1702559-supplementary.pdf]

Supplemental Material

Associating Increased Chemical Exposure to Hurricane Harvey In A Longitudinal Panel Using Silicone Wristbands.

S.M. Samon[1], D. Rohlman[2], L.G. Tidwell[1], P.D. Hoffman [1] A.O. Oluyomi [3,4], K.A. Anderson[1]

[1]Department of Environmental & Molecular Toxicology, Oregon State University, Corvallis, OR
[2]College of Public Health and Human Sciences, Oregon State University, Corvallis, OR
[3] Section of Epidemiology and Population Sciences, Department of Medicine, Baylor College of Medicine, Houston, TX
[4]Gulf Coast Center for Precision Environmental Health, Baylor College of Medicine, Houston, TX

Table of Contents

Page	Content
S2	Instrument Parameter & Analyte Quantification Explanatory paragraphs
S3	Table S1. Background Subtraction Applied
S4	Table S2. Screening Method: Detected Analytes & Chemical Use/Type Category
S11	Table S3. PAH Method: Target Analyte List
S13	Table S4. Participant Demographics
S14	Table S5. Summary Statistics for Covariate Analysis and Chemical Category Concentration: Participant Neighborhood
S15	Table S6. Summary Statistics for Covariate Analysis and Chemical Category Concentration: Race/Ethnicity
S16	Table S7. Summary Statistics for Covariate Analysis and Chemical Category Concentration: Area Deprivation Index Quartile
S17	Table S8. Summary Statistics for Covariate Analysis and Chemical Category Concentration: Home Flooding
S18	Table S9. Summary Statistics for Covariate Analysis and Chemical Category Concentration: Age
S19	Table S10. Summary Statistics for Covariate Analysis and Chemical Category Concentration: Gender
S20	Table S11. Summary Statistics Screening Method Categories
S20	Table S12. Summary Statistics 63 PAH Method Categories
S21	Figure S1. The Total Chemicals Detected Within The 1530 Screening Method Post Hurricane and At The Estimated Baseline By Chemical Classification.
S21	Figure S2. The Total PAHs Detected Within the 63 PAH Method Post Hurricane and at the Estimated Baseline by A) Ring Size, and B) Parent Vs. Alkylated Status.
S22	Figure S3. Composition of PAH Method Categories
S23	Table S13. Detection Frequencies in Hurricane Harvey Study & Other Non-disaster Studies

Instrument Parameters: GC-MS/MS - PAH Quantitative Method

A sample was loaded using an Agilent 4 mm liner and injected in pulsed splitless mode (320°C, 35 psi pulse pressure, 25 mL/min purge after 0.7 minutes) onto an Agilent Select PAH column (30 m × 0.25 mm × 0.15 um) with helium carrier gas (2 mL/min). The temperatures of the source and mass detector transfer line were set to 340°C, 320°C, and 300 °C respectively. The temperature profile started at 60°C (1 min), then ramped to 180°C (40°C/min), 230°C (3°C/min), 280°C (1.5°C/min), hold for 10 min, then ramped to 298°C (6°C/min), 350°C (16°C/min), and finally held at 350°C for 4 min (Anderson et al. 2015).

Analyte Quantification: PAH Quantitative Method

Target analyte data from GC-MS/MS was analyzed using MassHunter Quantitative Analysis v.B.06.00 SP1 build 6.0.388.1 (Agilent Corp. Wilmington, DE) software. Extraction surrogates (Table S1) were quantified relative to the internal standard (perylene$_{d12}$), and target analytes were quantified relative to the most appropriate surrogate (e.g. retention time). Positive analyte identification was based on retention time, peak shape, and the presence of at least one qualifier ion within ±30% of the expected value. PAH quantification occurred using an internal standard calibration with 7-9-point calibration curves (1 to 10,000 pg/uL) and coefficients of determination of >0.99. Limits of detection (LODs) were determined by running the 1 pg/uL standard seven times to calculate the standard deviation and 99% confidence interval with the Student's *t*-value. The limits of quantification (LOQs) were five times higher than the LODs (Anderson et al. 2015).

Instrument Parameters: GC-MS – 1,500 Screening Method

An Agilent DB-5MS column (30 m × 0.25 mm × 0.25 um) was operated in electron impact mode (70 eV) and the inlet pressure was locked to the retention time of chlorpyrifos (19.20 ± 0.20 min). Samples were loaded using an Agilent 4 mm liner and pulsed splitless injection. The temperatures of the MS source and quadruple, and detector transfer line were set to 150°C and 300 °C respectively. The pulse pressure was 25 psi (0.5 min) at a 3 mL/min purge and a 20 mL/min purge after 0.45 minutes. The temperature profile started at 70°C, then ramped to 200°C, 280°C, and 310°C, and finally held at 325°C (Bergmann et al. 2018).

Analyte Quantification: 1,500 Screening Method

As a high-throughput screen, the method used an automated mass spectral deconvolution and identification system (AMDIS v. 2.66, National Institute of Standards and Technology) coupled with deconvolution reporting software (DRS, Agilent). Target analyte quantified by AMDIS (≥60% library match) were manually reviewed to remove false positives. Positive analyte identification was based on previously reported data quality objectives to reduce false positives: retention time shifts ±0.75 min, peak responses greater than 3:1 signal-to-noise ratio, and at least one qualifier ion within the expected ratio with the quantifier ion (Bergmann et al. 2018; Dixon et al. 2019).

Table S1. Background Subtraction applied to all samples from both screening Method and PAH based off of a laboratory processing blank for the post-hurricane timepoint and a post-deployment cleaning blank for the estimated baseline timepoint.

	Post Hurricane		*Estimated Baseline*	
1,500 Screening Method	**Analyte**	**Background Subtracted (nmol/WB)**	No analytes detected in laboratory processing blank	
	Di-n-butyl phthalate	1.8		
	Diethyl phthalate	0.99		
	Diisobutyl phthalate	0.097		
	Butylated hydroxytoluene	10		
	Bis(2-ethylhexyl) phthalate	0.28		
	1-methylnaphthalene	0.098		
PAH Quantitative Method	**Analyte**	**Background Subtracted (nmol/WB)**	**Analyte**	**Background Subtracted (nmol/WB)**
	Naphthalene	0.281	Naphthalene	0.101
	2-methylnaphthalene	0.158	2-methylnaphthalene	0.0483
	1-methylnaphthalene	0.111	1-methylnaphthalene	0.0534
	1,6 and 1,3-dimethyl-naphthalene	0.0800	2-ethylnaphthalene	0.0305
	1,4-dimethyl-naphthalene	0.0157		
	1,5-dimethyl-naphthalene	0.0658		
	1,2-dimethyl-naphthalene	0.00769		
	Fluorene	0.00997		
	Dibenzothiophene	0.00350		
	Phenanthrene	0.0262		
	2-Methylphenanthrene	0.0149		

Table S2. Analytes detected utilizing the screening method and corresponding CAS number, detection frequencies, primary type/use category utilized, and other type/use cauterizations. The primary type/use category denoted here is the categorization utilized for statistical analysis.

Analyte	CAS Number	T1 Frequency	T2 Frequency	Primary Category	Other Type/Use Categorizations
Dibenzofuran	132-64-9	0.0	1.0	Dioxins/Furans	
Triphenyl phosphate	115-86-6	85.9	74.7	Flame Retardants	Industrial, plasticizer, organophosphate ester (OPE)
Tris(2-chloroisopropyl) phosphate	26248-87-3	41.4	71.7	Flame Retardants	Industrial, plasticizer, organophosphate ester (OPE)
Tris(2-ethylhexyl) phosphate	78-42-2	42.4	4.0	Flame Retardants	Industrial, plasticizer, pesticide organophosphate ester (OPE)
Tributyl phosphate	126-73-8	22.2	13.1	flame retardant	Industrial, plasticizer, organophosphate ester (OPE)
PBDE 47	5436-43-1	29.3	2.0	Flame Retardants	Polybrominated diphenyl ether (PBDE)
PBDE 49	243982-82-3	9.1	2.0	Flame Retardants	Polybrominated diphenyl ether (PBDE)
Tricresylphosphate, meta-	563-04-2	7.1	4.0	Flame Retardants	Industrial, plasticizer, organophosphate ester (OPE)
Tricresylphosphate, ortho-	78-30-8	6.1	2.0	Flame Retardants	Industrial, plasticizer, organophosphate ester (OPE)
TCEP	115-96-8	3.0	3.0	Flame Retardants	Industrial, plasticizer, pesticide organophosphate ester (OPE)
PBDE 99	60348-60-9	3.0	1.0	Flame Retardants	Polybrominated diphenyl ether (PBDE)
PBDE 119	189084-66-0	3.0	0.0	Flame Retardants	Polybrominated diphenyl ether (PBDE)
PBDE 153	68631-49-2	1.0	0.0	Flame Retardants	Polybrominated diphenyl ether (PBDE)
PBDE 100	189084-64-8	1.0	0.0	Flame Retardants	Polybrominated diphenyl ether (PBDE)
PBDE 118	446254-80-4	0.0	1.0	Flame Retardants	Polybrominated diphenyl ether (PBDE)
PBDE 103	446254-67-7	1.0	0.0	Flame Retardants	Polybrominated diphenyl ether (PBDE)
Butylated hydroxytoluene	128-37-0	77.8	85.9	Industrial	Food additive, antioxidant
Benzothiazole	95-16-9	50..5	39.4	Industrial	Benzothiazoles, food additive
2,4-di-tert-butylphenol	96-76-4	70.7	12.1	Industrial	Phenol, antioxidant
Eugenol	97-53-0	18.2	9.1	Industrial	Personal Care, Pesticide, Pharmacological
4-tert-butylphenol	98-54-4	8.1	13.1	Industrial	Phenol, food additive

4-chloro-3,5-dimethylphenol	88-04-0	8.1	12.1	Industrial	Phenol, pharmacological
Biphenyl	92-52-4	16.2	2.0	Industrial	PAH, food additive, pesticide
Drometrizole	2440-22-4	8.1	7.1	Industrial	Triazole, plasticizer
Triethyl phosphate	78-40-0	5.1	7.1	Industrial	Pesticide, plasticizer, flame Retardants, organophosphate ester (OPE)
4-Chlorophenyl isocyanate	104-12-1	7.1	4.0	Industrial	Pesticide, pharmaceutical
Diuron Metabolite [3,4-Dichlorophenyl isocyanate]	102-36-3	6.1	4.0	Industrial	Pesticide
3-tert-butylphenol	585-34-2	8.1	1.0	Industrial	Phenol, fragrance
Benzyl alcohol	100-51-6	4.0	4.0	Industrial	Personal Care, fragrance, food additive
3-Chloroaniline	108-42-9	4.0	3.0	Industrial	Pesticide
Guaiacol	90-05-1	2.0	4.0	Industrial	Phenol, food additive, antioxidant
Carvone	99-49-0	2.0	3.0	Industrial	Personal Care, pesticide, food additive, fragrance
2,6-Di-tert-butylphenol	128-39-2	5.1	0.0	Industrial	Phenol, antioxidant
2-Methylphenol	95-48-7	3.0	1.0	Industrial	Phenol, pesticide
Tri-p-tolyl phosphate	78-32-0	2.0	2.0	Industrial	Flame retardant, plasticizer, organophosphate ester (OPE)
4-isopropylphenol	99-89-8	3.0	1.0	Industrial	Personal Care, food additive
Quinoline	91-22-5	3.0	0.0	Industrial	Quinoline
2,4-Dichlorophenol	120-83-2	1.0	1.0	Industrial	Pesticide, phenol
Azobenzene	103-33-3	2.0	0.0	Industrial	Pesticide
Tolyltriazole [1H-Benzotriazole, 4-methyl-]	29878-31-7	0.0	2.0	Industrial	Triazole
2,4-bis(alpha,alpha-dimethylbenzyl)phenol	2772-45-4	1.0	1.0	Industrial	Phenol
2-isopropylphenol	88-69-7	1.0	1.0	Industrial	Phenol. food additive
2-methoxy-4-methylphenol	93-51-6	1.0	1.0	Industrial	Phenol. food additive, fragrance
1-Naphthylamine	134-32-7	0.0	1.0	Industrial	
4-Methylphenol	106-44-5	0.0	1.0	Industrial	Phenol, food additive, personal care product
3,5-Dichloroaniline	626-43-7	0.0	1.0	Industrial	Pesticide
3,4-Dichloroaniline	95-76-1	0.0	1.0	Industrial	Pesticide
Phthalimide	85-41-6	0.0	1.0	Industrial	Pesticide
2,6-dimethylphenol	576-26-1	1.0	0.0	Industrial	Phenol. food additive, fragrance

2,3,6-trimethylphenol	2416-94-6	1.0	0.0	Industrial	Phenol. food additive, fragrance
2-Chlorosyringaldehyde	76341-69-0	0.0	1.0	Industrial	
Indole	120-72-9	0.0	1.0	Industrial	Food additive, fragrance
Benzotriazole	95-14-7	0.0	1.0	Industrial	Triazole
1-methylnaphthalene	90-12-0	69.7	29.3	PAH	Industrial, alkyl PAH, food additive
Anthracene	120-12-7	75.8	19.2	PAH	Parent PAH, pesticides, industrial
naphthalene	91-20-3	34.3	33.3	PAH	Parent PAH, industrial, pesticide
2-methylphenanthrene	2531-84-2	30.3	8.1	PAH	Alkyl PAH
1-methylphenanthrene	832-69-9	25.3	2.0	PAH	Alkyl PAH
Pyrene	129-00-0	20.2	6.1	PAH	Industrial, parent PAH
3,6-dimethylphenanthrene	1576-67-6	18.2	1.0	PAH	Alkyl PAH
fluorene	86-73-7	14.1	2.0	PAH	Parent PAH
2,6-dimethylnaphthalene	581-42-0	10.1	0.0	PAH	Alkyl PAH
retene	483-65-8	7.1	2.0	PAH	Alkyl PAH
1,6-dimethylnaphthalene	575-43-9	8.1	0.0	PAH	Alkyl PAH
1-Hydroxynaphthalene	90-15-3	0.0	6.1	PAH	Industrial, oxygenated PAH (OPAH)
acenaphthylene	208-96-8	3.0	1.0	PAH	Parent PAH
phenanthrene	85-01-8	2.0	2.0	PAH	Parent PAH, industrial
2-methylnaphthalene	91-57-6	1.0	2.0	PAH	Industrial, alkyl PAH
acenaphthene	83-32-9	3.0	0.0	PAH	Parent PAH, industrial
2,3-dimethylanthracene	613-06-9	3.0	0.0	PAH	Alkyl PAH
9-Fluorenone	486-25-9	2.0	0.0	PAH	Industrial, oxygenated PAH
1,2-dimethylnaphthalene	573-98-8	2.0	0.0	PAH	Alkyl PAH
1-methylpyrene	2381-21-7	2.0	0.0	PAH	Alkyl PAH
2,6-diethylnaphthalene	59919-41-4	2.0	0.0	PAH	Alkyl PAH
benz[a]anthracene	56-55-3	1.0	0.0	PAH	Parent PAH
benzo[a]pyrene	50-32-8	0.0	1.0	PAH	Parent PAH
fluoranthene	206-44-0	1.0	0.0	PAH	Parent PAH
9-methylanthracene	779-02-2	1.0	0.0	PAH	Alkyl PAH
2-ethylnaphthalene	939-27-5	1.0	0.0	PAH	Alkyl PAH
benzo[b]fluorene	243-17-4	1.0	0.0	PAH	Parent PAH

PCB 88	55215-17-3	2.0	0.0	PCB	Industrial
PCB 93	73575-56-1	1.0	0.0	PCB	Industrial
PCB 96	73575-54-9	0.0	1.0	PCB	Industrial
PCB 139	56030-56-9	1.0	0.0	PCB	Industrial
PCB 168	59291-65-5	1.0	0.0	PCB	Industrial
Galaxolide	1222-05-5	94.9	97.0	Personal Care	Fragrance
Tonalide	1506-02-1	84.8	93.9	Personal Care	Fragrance
Lilial	80-54-6	82.8	86.9	Personal Care	Fragrance
Benzophenone	119-61-9	85.9	80.8	Personal Care	Food additive, fragrance, industrial
Ethylene brassylate	105-95-3	61.6	85.9	Personal Care	Macrolide, food additive, fragrance
Benzyl salicylate	118-58-1	51.5	92.9	Personal Care	Food additive, fragrance
Amyl cinnamal	122-40-7	66.7	61.6	Personal Care	Food additive, fragrance
Butylated hydroxyanisole	25013-16-5	50.5	66.7	Personal Care	Food additive, antioxidant
b-Ionone	79-77-6	48.5	59.6	Personal Care	Food additive, fragrance
Linalool	78-70-6	29.3	32.3	Personal Care	Food additive, fragrance, pesticide
d-Limonene	5989-27-5	34.3	11.1	Personal Care	Fragrance, industrial, terpene
Triclosan	3380-34-5	27.3	13.1	Personal Care	Pharmacological, phenol
b-citronellol	106-22-9	10.1	25.3	Personal Care	Fragrance
Cinnamaldehyde	104-55-2	15.2	16.2	Personal Care	Fragrance, pesticide, pharmacological
Musk Ketone	81-14-1	11.1	16.2	Personal Care	Aromatic ketone, fragrance
Exaltolide [15-Pentadecanolide]	106-02-5	16.2	10.1	Personal Care	Macrolide, food additive, fragrance
a-Ionone	127-41-3	19.2	7.1	Personal Care	Food additive, fragrance
Celestolide	13171-00-1	13.1	6.1	Personal Care	Indane, fragrance
Hydroxy-citronellal	107-75-5	8.1	11.1	Personal Care	Fragrance
Cashmeran	33704-61-9	9.1	7.1	Personal Care	Fragrance
Benzyl cinnamate	103-41-3	9.1	3.0	Personal Care	Fragrance
Citral A	5392-40-5	3.03	6.1	Personal Care	Food additive, fragrance
Citral B	5392-40-5	1.0	6.1	Personal Care	Food additive, fragrance
Geraniol	106-24-1	1.0	3.0	Personal Care	Food additive, fragrance, pesticide, pharmacological, terpenoid
Isoeugenol	97-54-1	2.0	1.0	Personal Care	Food additive, fragrance, phenylpropanoid

Farnesol III	4602-84-0	1.0	2.0	Personal Care	Fragrance
Phantolide	15323-35-0	2.0	0.0	Personal Care	Fragrance
Cyclopentadecanone	502-72-7	1.0	1.0	Personal Care	Fragrance
Methyleugenol	93-15-2	1.0	1.0	Personal Care	Food additive, fragrance, phenylpropanoid
Lyral	31906-04-4	1.0	1.0	Personal Care	Fragrance
2,3,5-trimethylphenol	697-82-5	2.0	0.0	Personal Care	Industrial
Farnesol IV	4602-84-0	1.0	1.0	Personal Care	Fragrance
Farnesol I	4602-84-0	1.0	0.0	Personal Care	Fragrance
Farnesol II	4602-84-0	1.0	0.0	Personal Care	Fragrance
Methyl 2-octynoate	111-12-6	0.0	1.0	Personal Care	Fragrance
Propenyl guaethol	94-86-0	1.0	0.0	Personal Care	
Musk amberette	83-66-9	1.0	0.0	Personal Care	
N,N-Diethyl-m-toluamide	134-62-3	83.8	89.9	Pesticide	Personal care
Benzyl benzoate	120-51-4	44.4	60.6	Pesticide	Pharmacological, fragrance
Permethrin	52645-53-1	46.5	53.5	Pesticide	Pharmacological, pyrethroid ester insecticide
Coumarin	91-64-5	14.1	56.6	Pesticide	Fragrance
Permethrin II	61949-76-6	29.3	29.3	Pesticide	Pharmacological, pyrethroid ester insecticide
Bifenthrin	82657-04-3	35.4	18.2	Pesticide	
Piperonyl butoxide	51-03-6	15.2	11.1	Pesticide	
Ethofenprox	80844-07-1	12.1	14.1	Pesticide	
Cypermethrin-2	52315-07-8	6.1	5.1	Pesticide	Pharmacological, pyrethroid ester insecticide
o-Phenylphenol	90-43-7	4.0	5.1	Pesticide	Industrial, phenol, antioxidant
Pentachloroanisole	1825-21-4	7.1	1.0	Pesticide	Industrial
gamma-Chlordane	5103-74-2	3.0	4.0	Pesticide	
Chlorpyrifos	2921-88-2	4.0	0.0	Pesticide	Pharmacological
Cyhalothrin I (lambda)	68085-85-8	4.0	0.0	Pesticide	Organophosphate pesticide
4,4'-DDT	50-29-3	3.0	0.0	Pesticide	Organochlorine insecticide
alpha-Chlordane	5103-71-9	2.0	1.0	Pesticide	
trans-Nonachlor	39765-80-5	1.0	2.0	Pesticide	Organochlorine insecticide
d-(cis-trans)-Phenothrin-I	26002-80-2	1.0	2.0	Pesticide	Pharmacological, pyrethroid ester insecticide
Pyriproxyfen	95737-68-1	3.0	0.0	Pesticide	Pyridine

Cypermethrin-3	52315-07-8	2.0	1.0	Pesticide	Pharmacological, pyrethroid ester insecticide
Promecarb artifact [5-isopropyl-3-methylphenol]	3-3-3228	2.0	1.0	Pesticide	
Isobornyl thiocyanoacetate	115-31-1	2.0	0.0	Pesticide	
Methoprene I	40596-69-8	1.0	1.0	Pesticide	
Tetramethrin I	7696-12-0	1.0	1.0	Pesticide	Pyrethroid ester insecticide
Azoxystrobin	131860-33-8	2.0	0.0	Pesticide	
Fenobucarb	3766-81-2	0.0	2.0	Pesticide	
Cyhalothrin (Gamma)	91465-08-6	2.0	0.0	Pesticide	Pyrethroid ester insecticide
Methoprene II	65733-16-6	1.0	1.0	Pesticide	Pharmacological
4,4'-DDD	72-54-8	1.0	0.0	Pesticide	Organochlorine insecticide
4,4'-DDE	72-55-9	1.0	0.0	Pesticide	Organochlorine insecticide
Dichlorvos	62-73-7	0.0	1.0	Pesticide	Organophosphate pesticide
Malathion	121-75-5	0.0	1.0	Pesticide	Organophosphate pesticide, pharmacological
Methoxychlor	72-43-5	1.0	0.0	Pesticide	Organochlorine insecticide pharmacological
Promecarb	2631-37-0	0.0	1.0	Pesticide	
Pentachloronitrobenzene	82-68-8	1.0	0.0	Pesticide	
Tebuconazole	107534-96-3	0.0	1.0	Pesticide	Triazole
Deltamethrin	52918-63-5	1.0	0.0	Pesticide	Pyrethroid ester insecticide
Pyrimethanil	53112-28-0	0.0	1.0	Pesticide	Aminopyrimidine
Dimethametryn	22936-75-0	1.0	0.0	Pesticide	Triazine
Tetrachlorvinphos	961-11-5	1.0	0.0	Pesticide	Organophosphate pesticide, organochlorine insecticide
Oxycarboxin	5259-88-1	1.0	0.0	Pesticide	Anilide
Bromopropylate	18181-80-1	0.0	1.0	Pesticide	
Hydroprene	41096-46-2	0.0	1.0	Pesticide	
Fipronil, Desulfinyl-	205650-65-3	1.0	0.0	Pesticide	Pyrazole
Fipronil-sulfide	120067-83-6	1.0	0.0	Pesticide	Pyrazole
Fipronil	120068-37-3	1.0	0.0	Pesticide	Pyrazole, pharmacological

Mepanipyrim	110235-47-7	1.0	0.0	Pesticide	Aminopyrimidine
Cinerin I	25402-06-6	0.0	1.0	Pesticide	Pyrethroid ester insecticide
Tetramethrin II	999050-03-2	1.0	0.0	Pesticide	Pyrethroid ester insecticide
d-(cis-trans)-Phenothrin-II	999034-03-6	0.0	1.0	Pesticide	Pyrethroid ester insecticide
Cyfluthrin II	999028-03-4	1.0	0.0	Pesticide	Pyrethroid ester insecticide
Cypermethrin-4	52315-07-8	0.0	1.0	Pesticide	Pharmacological, pyrethroid ester insecticide
XMC (3,4-Dimethylphenyl N-methylcarbamate)	10-7-2425	0.0	1.0	Pesticide	
Caffeine	58-08-2	39.4	50.5	Pharmacological	Personal care products
Thymol	89-83-8	5.1	3.0	Pharmacological	Phenol, food additive
Butyl benzyl phthalate	85-68-7	97.0	98.0	Phthalate	Industrial, plasticizer
Di-n-butyl phthalate	84-74-2	93.9	100	Phthalate	Industrial, plasticizer
Diisobutyl phthalate	84-69-5	79.8	99.0	Phthalate	Industrial, plasticizer
Diethyl phthalate	84-66-2	89.9	80.8	Phthalate	Industrial, plasticizer, personal care product
Di-n-nonyl phthalate	84-76-4	50.5	83.8	Phthalate	Industrial, plasticizer
Bis(2-ethylhexyl) phthalate	117-81-7	22.2	92.9	Phthalate	Industrial, plasticizer
Di-n-hexyl phthalate	84-75-3	18.2	13.1	Phthalate	Industrial, plasticizer
Dicyclohexyl phthalate	84-61-7	10.1	20.2	Phthalate	Industrial, plasticizer
Di-n-octyl phthalate	117-84-0	6.1	16.2	Phthalate	Industrial, plasticizer
Dimethyl phthalate	131-11-3	5.1	17.2	Phthalate	Industrial, plasticizer
Di-n-propyl phthalate	131-16-8	0.0	2.0	Phthalate	Industrial, plasticizer

Table S3. The PAH analytical method included 63 target analytes, for which the CAS number, molecular weight (MW), instrument limits of detection (LOD) and quantitation (LOQ), and detection frequencies for both time points are provided.

Target Analyte	MW (g/mole)	CAS Number	Parent or Alkylated	Ring Size	Instru. LOD (pmol/uL)	Instru. LOQ (pmol/uL)	Detection Frequency Post-hurricane	Detection Frequency Estimated Baseline
Naphthalene	128.17	91-20-3	Parent	2	0.33	1.00	99.0	100
2-Methylnaphthalene	142.20	91-57-6	Alkylated	2	1.04	5.20	99.0	100
1-Methylnaphthalene	142.20	90-12-0	Alkylated	2	0.28	1.39	98.0	100
2-Ethylnaphthalene	156.09	939-27-5	Alkylated	2	0.97	4.84	84.8	69.7
2,6-Dimethylnaphthalene	156.22	581-42-0	Alkylated	2	0.89	4.43	13.1	23.2
1,3- and 1,6-Dimethylnaphthalene	--	575-41-7, 575-43-9	Alkylated	2	0.81	4.05	89.9	80.8
1,4-Dimethylnaphthalene	156.22	571-58-4	Alkylated	2	1.24	6.22	64.6	47.5
1,5-Dimethylnaphthalene	156.22	571-61-9	Alkylated	2	1.19	5.93	55.6	43.4
1,2-Dimethylnaphthalene	156.22	573-98-8	Alkylated	2	0.94	4.70	69.7	52.5
1,8-Dimethylnaphthalene	156.22	569-41-5	Alkylated	2	0.83	4.15	0	0
2,6-Diethylnaphthalene	184.27	59919-41-4	Alkylated	2	0.81	4.06	3.0	0
Acenaphthylene	152.19	208-96-8	Parent	3	2.33	11.6	35.4	22.2
Acenaphthene	154.20	83-32-9	Parent	3	1.07	5.35	64.6	9.1
Fluorene	166.22	86-73-7	Parent	3	0.79	3.97	81.8	92.9
Dibenzothiophene	184.26	132-65-0	Parent	3	0.24	1.20	91.9	100
Phenanthrene	178.23	85-01-8	Parent	3	0.46	2.31	96.0	100
Anthracene	178.23	120-12-7	Parent	3	1.05	5.23	30.3	19.2
2-Methylphenanthrene	192.25	2531-84-2	Alkylated	3	0.39	1.93	88.9	100
2-Methylanthracene	192.25	613-12-7	Alkylated	3	0.47	2.36	12.1	11.1
1-Methylphenanthrene	192.25	832-69-9	Alkylated	3	1.06	5.32	92.9	100
9-Methylanthracene	192.25	779-02-2	Alkylated	3	0.87	4.37	0	0
3,6-Dimethylphenanthrene	206.28	1576-67-6	Alkylated	3	0.42	2.08	78.8	75.8
2,3-Dimethylanthracene	206.28	613-06-9	Alkylated	3	0.34	1.71	39.4	0
9,10-Dimethylanthracene	206.28	781-43-1	Alkylated	3	0.85	4.23	0	0
Retene	234.33	483-65-8	Alkylated	3	0.84	4.19	99.0	100
Fluoranthene	202.26	206-44-0	Parent	4	0.54	2.72	99.0	97.0
Pyrene	202.25	129-00-0	Parent	4	0.42	2.09	99.0	100
Benzo[a]fluorene	216.23	238-84-6	Parent	4	1.67	5.00	25.3	7.1
Benzo[b]fluorene	216.23	243-17-4	Parent	4	1.67	5.00	3.0	2.0
Benzo[c]fluorene	216.23	205-12-9	Parent	4	0.30	1.50	4.0	2.0

Benz[a]anthracene	228.29	56-55-3	Parent	4	0.75	3.77	34.3	18.2
Triphenylene	228.29	217-59-4	Parent	4	0.41	2.04	57.6	31.3
Chrysene	228.28	218-01-9	Parent	4	0.50	2.49	56.6	29.3
1-Methylpyrene	216.28	2381-21-7	Alkylated	4	0.38	1.90	100	92.9
6-Methylchrysene	242.31	1705-85-7	Alkylated	4	0.89	4.44	4.0	1.0
5-Methylchrysene	242.31	3697-24-3	Alkylated	4	1.67	5.00	2.0	0
7,12-Dimethylbenz[a]anthracene	256.34	57-97-6	Alkylated	4	0.94	4.71	11.1	6.1
Cyclopenta[c,d]pyrene	229.27	27208-37-3	Parent	5	0.53	2.67	12.1	5.1
Benzo[b]fluoranthene	252.30	205-99-2	Parent	5	0.37	1.85	64.6	43.4
Benzo[k]fluoranthene	252.30	207-08-9	Parent	5	0.53	2.63	37.4	9.1
Benzo[j]fluoranthene	252.30	205-82-3	Parent	5	0.56	2.79	30.3	8.1
Benz[j] and [e]aceanthrylene	--	202-33-5, 199-54-2	Parent	5	1.67	5.00	0	0
Benzo[e]pyrene	252.30	192-97-2	Parent	5	0.71	3.53	60.6	24.2
Benzo[a]pyrene	252.30	50-32-8	Parent	5	1.18	5.90	23.2	5.1
Perylene	252.32	198-55-0	Parent	5	1.01	5.47	0	1.0
Picene	278.35	213-46-7	Parent	5	0.74	3.72	3.0	1.0
Indeno[1,2,3-c,d]pyrene	276.33	193-39-5	Parent	6	0.26	1.32	17.2	5.1
Dibenzo[a,h]pyrene	278.35	53-70-3	Parent	6	1.02	5.11	0	0
Benzo[ghi]perylene	276.33	191-24-2	Parent	6	0.34	1.71	93.9	44.4
Anthanthrene	276.33	191-26-4	Parent	6	0.33	1.65	12.1	0
Naphtho[1,2-b] fluoranthene	302.36	5385-22-8	Parent	6	1.67	5.00	5.1	1.0
Naphtho[2,3-j] and [1,2-k] fluoranthene	--	205-83-4, 238-04-0	Parent	6	1.67	5.00	3.0	1.0
Dibenzo[a,e]fluoranthene	302.37	5385-75-1	Parent	6	0.47	2.36	3.0	1.0
Dibenzo[a,l]pyrene	302.37	191-30-0	Parent	6	0.48	2.41	0	1.0
Naphtho[2,3-k] fluoranthene	302.37	207-18-1	Parent	6	1.67	5.00	0	0
Naphtho[2,3-e] pyrene	302.37	193-09-9	Parent	6	1.67	5.00	0	0
Dibenzo[a,e]pyrene	302.37	192-65-4	Parent	6	6.44	32.2	0	0
Dibenzo[e,l]pyrene	302.36	192-51-8	Parent	6	1.67	5.00	5.1	1.0
Naphtho[2,3-a] pyrene	302.36	196-42-9	Parent	6	1.67	5.00	0	0
Benzo[b]perylene	302.36	197-70-6	Parent	6	1.67	5.00	0	0
Dibenzo[a,i]pyrene	302.36	189-55-9	Parent	6	1.42	7.10	0	0
Dibenzo[a,h]pyrene	302.36	189-64-0	Parent	6	0.52	2.60	0	0
Coronene	300.35	191-07-1	Parent	7	0.70	3.49	81.8	2.0

Table S4. Houston-3H longitudinal panel participant demographics.

Characteristics	N	%
Gender		
Female	70	70.7
Male	29	28.3
Age		
Children (9-17)	7	7.1
Adult (18-64)	66	66.7
Senior (>64)	26	29.3
Race/Ethnicity		
Asian	11	11.1
Black/African American	31	31.3
Latino	16	16.2
White	38	38.4
Other/Multiracial	3	3.0
Area Deprivation Index Quartile (ADI)		
First Quartile	34	34.3
Second Quartile	24	24.2
Third Quartile	25	25.2
Fourth Quartile	16	16.2
Neighborhood		
Addick's	18	18.2
Bellaire-Meyerland	26	26.3
Baytown	28	28.3
East Houston	15	15.1
Unclassified	12	12.1
Home Flooding		
Home Flooded	37	37.4
Home Flooded & Moved	44	44.4
Home Did Not Flood	18	18.2

Table S5. Summary statistics for the difference in the sum concentration of exposures for the 1,500 screening method, 63 PAH method, and their associated chemical categories by participant neighborhood. Differences were calculated by subtracting individual estimated baseline concentrations from the paired post-hurricane concentration. A positive value indicates a higher concentration post-hurricane.

	**Min, Median, Max (nmol/WB)**					**Mean (nmole/WB), SE**						
Target Analyte	A	BM	B	EH	U	A	BM	B	EH	U	Kruskal Wallis Statistic	P value
**Σ [1,500 screen]**	-4300, 500, 2000	-2000, 330, 2300	-2500, 870, 2700	-3000, 410, 3000	-2000, -470, 1600	300, 330	270, 210	550, 250	370, 370	-200, 350	3.9	0.42
Σ [PCPs]	-450, 260, 1000	-2700, 150, 1600	-1300, 500, 1600	-1500, 100, 1400	-2300, 220, 910	310, 94	120, 140	410, 120	44, 200	110, 250	6.1	0.19
Σ [Phthalates]	-4700, 100, 830	-1200, 84, 870	-2900, 200, 950	-3200, 230, 810	-1200, 68, 530	-210, 290	-76, 120	-40, 170	-99, 250	-240, 200	2.5	0.64
Σ [Pesticides]	-1500, 88, 490	-940, 150, 580	-810, 9.1, 1000	-820, 280, 1300	-3200, 32, 1200	53, 99	73, 78	3.4, 80	290, 130	-220, 310	7.0	0.14
Σ [Flame Retardant]	-100, 15, 130	-79, 23, 760	-6.0, 32, 410	-4.0, 22, 360	6.0, 34, 130	21, 10	67, 31	64, 18	71, 28	50, 13	3.0	0.56
Σ [Pharmaceutical]	-100, 0, 460	-82, 0, 690	-20, 0, 390	-60, 0, 120	87, 0, 120	56, 30	58, 29	55, 19	6.7, 11	15, 15	4.4	0.36
Σ [Industrial]	6.2, 43, 160	-91, 27, 120	-90, 44, 260	-7.4, 15, 150	5.1, 34, 370	60, 12	21, 7.8	55, 11	36, 11	84, 33	10	0.035
**Σ [63 PAH method]**	-15.7, 1.42, 5.54	-7.71, 1.22, 26.3	-15.4, 0.200, 33.7	-14.3, 0.200, 162	-217, -0.132, 4.43	-0.588, 1.16	1.84, 1.21	1.90, 1.60	16.0, 10.9	-19.6, 18.0	5.97	0.202
Σ [2-ring PAH]	-4.03, 0.241, 2.37	-10.6, -0.408, 15.1	-15.9, -0.972, 18.3	-7.77, -0.972, 18.3	-214, -0.248, 1.33	0.0232, 0.364	-0.0931, 0.797	-0.681, 1.03	11.5, 11.2	-19.6, 17.8	3.44	0.487
Σ [3-ring PAH]	-14.2, 0.669, 3.07	-1.01, 0.952, 10.6	-1.51, 0.280, 41.4	-5.03, 0.114, 41.9	-4.34, -0.182, 3.22	-0.870, 0.928	1.61, 0.559	2.07, 1.49	3.47, 2.95	-0.277, 0.567	5.04	0.284
Σ [4-ring PAH]	-0.863, 0.106, 1.89	-0.294, 0.250, 0.899	-0.461, 0.337, 1.91	-2.76, 0.353, 10.9	-0.810, 0.0874, 2.98	0.147, 0.147	0.263, 0.0545	0.434, 0.0987	1.14, 0.790	0.240, 0.276	6.90	0.141
Σ [5-ring PAH]	-0.0237, 0.0245, 0.347	-0.171, 0.00955, 0.118	-0.129, 0, 0.194	-2.31, 0.0348, 0.353	-0.0334, 0, 0.337	0.0447, 0.0204	0.0157, 0.0113	0.0314, 0.0143	-0.111, 0.161	0.0452, 0.0287	1.17	0.883
Σ [6&7-ring PAH]	0.00302, 0.0169, 0.530	0, 0.0266, 0.391	0, 0.0242, 0.263	-0.597, 0.0230, 0.121	-0.0123, 0.0145, 0.180	0.0664, 0.0317	0.0411, 0.0143	0.0410, 0.0101	-0.0210, 0.0438	0.0278, 0.0144	3.70	0.448
Σ [parent PAH]	-2.46, 0.290, 3.74	-5.65, 1.15, 7.13	-14.2, 0.569, 27.7	-8.70, 0.171, 44.3	-214, -0.137, 2.34	0.204, 0.393	1.14, 0.470	1.79, 1.32	7.14, 4.01	-19.3, 17.8	6.99	0.136
Σ [alkylated PAH]	-13.9, 0.204, 1.84	-4.63, -0.310, 20.5	-5.39, -0.144, 5.92	-5.090.130, 117	-6.13, -0.0707, 1.20	-0.928, 0.866	0.567, 0.865	-0.149, 0.423	8.42, 7.80	-0.527, 0.592	1.69	0.793

A: Addick's, B: Baytown, BM: Bellaire-Meyerland, U: Unclassified, EH: East Houston; * **(p < 0.008)**

Table S6. Summary statistics for the difference in the sum concentration of exposures for the 1,500 screening method, 63 PAH method, and their associated chemical categories by race/ethnicity. Differences were calculated by subtracting individual estimated baseline concentrations from the paired post-hurricane concentration. A positive value indicates a higher concentration post-hurricane.

	**Min, Median, Max (nmol/WB)**				**Mean (nmole/WB), SE**					
Target Analyte	Asian	White	Latino	Black	Asian	White	Latino	Black	Kruskal Wallis Statistic	P value
**Σ [1,500 screen]**	-1300, 800, 1900	-4300, 320, 2300	-670, 401, 2200	-3100, 650, 3000	420, 310	130, 210	560, 220	260, 270	0.91	0.82
Σ [PCPs]	-320, 340, 1000	-2700, 150, 1600	-1500, 220, 780	-1300, 450, 1400	360, 110	120, 125	140, 140	290, 130	3.0	0.39
Σ [Phthalates]	-1100, 130, 530	-4700, 140, 834	-1700, 200, 870	-3200, 130, 950	-160, 190	-150, 150	-59, 180	-130, 183	0.58	0.90
Σ [Pesticides]	-940, 85, 580	-3200, 54, 1200	-770, 280, 1300	-1200, 56, 800	62, 120	5.9, 110	320, 120	-40, 84	5.9	0.12
Σ [Flame Retardant]	5.6, 34, 230	-100, 21, 760	-4.5, 20, 410	-6.2, 25, 360	57, 20	46, 21	93, 31	45, 14	3.7	0.29
Σ [Pharmaceutical]	-7.9, 0, 100	-87, 0, 685	-110, 0, 270	-29, 0, 390	21, 12	58, 23	22, 21	45, 17	1.2	0.76
Σ [Industrial]	-5.0, 53, 370	-91, 24, 270	-7.4, 26, 105	-90, 40, 150	77, 31	42, 10	33, 8.3	44, 8.1	3.1	0.38
**Σ [63 PAH method]**	-217, 0.816, 26.3	-15.7, 1.34, 9.94	-6.21, 0.362, 42.8	-15.4, 0.982, 162	-17.8, 20.1	0.512, 0.723	3.24, 2.79	7.23, 5.42	0.219	0.974
Σ [2-ring PAH]	-214, 0.199, 15.1	-10.6, -0.299, 5.68	-7.77, -0.00460, 1.42	-15.9, -0.913, 166	-19.4, 19.6	-0.405, 0.404	-0.653, 0.546	5.20, 5.51	0.639	0.888
Σ [3-ring PAH]	-2.36, 0.990, 10.6	-14.2, 0.801, 9.72	-5.19, 0.0705, 41.9	-5.03, 0.249, 41.4	1.35, 1.01	0.532, 0.531	2.91, 2.74	1.65, 1.40	1.81	0.613
Σ [4-ring PAH]	-0.231, 0.186, 0.569	-0.863, 0.233, 2.98	-1.35, 0.182, 10.9	-2.76, 0.353, 2.52	0.183, 0.0651	0.291, 0.104	0.951, 0.709	0.393, 0.163	1.74	0.627
Σ [5-ring PAH]	-0.0307, 0, 0.0914	-0.171, 0.0222, 0.347	-0.521, 0.00786, 0.242	-2.31, 0.0283, 0.353	0.0173, 0.0105	0.0401, 0.0145	0.0127, 0.0267	-0.0278, 0.0776	1.08	0.782
Σ [6&7-ring PAH]	-0.0123, 0.0176, 0.180	0, 0.0239, 0.530	-0.157, 0.0308, 0.0836	-0.597, 0.0249, 0.263	0.0282, 0.0155	0.0539, 0.0178	0.0231, 0.0130	0.0219, 0.0227	3.86	0.278
Σ [parent PAH]	-214, 0.647, 5.55	-5.65, 0.780, 7.13	-2.96, 0.0886, 39.0	-14.2, 0.300, 44.3	-19.6, 19.6	0.712, 0.342	-2.69, 2.34	3.37, 2.0	0.547	0.908
Σ [alkylated PAH]	-2.41, 0.00133, 20.5	-13.9, 0.0608, 4.17	-3.79, -0.0715, 6.39	-6.13, -0.0364, 117	1.72, 1.91	-0.408, 0.454	0.255, 0.623	3.60, 3.81	0.0936	0.993

Excluding 3 participants who identified as multiracial; *** (p < 0.008)**

Table S7. Summary statistics for the difference in the sum concentration of exposures for the 1,500 screening method, 63 PAH method, and their associated chemical categories by Area Deprivation Index (ADI) quartiles. Differences were calculated by subtracting individual estimated baseline concentrations from the paired post-hurricane concentration. A positive value indicates a higher concentration post-hurricane.

	**Min, Median, Max (nmol/WB)**				**Mean (nmole/WB), SE**					
Target Analyte	First Quartile	Second Quartile	Third Quartile	Fourth Quartile	First Quartile	Second Quartile	Third Quartile	Fourth Quartile	Kruskal Wallis Statistic	P value
**Σ [1,500 screen]**	-2000, 480, 2300	-4300, 170, 1600	-2500, 710, 2700	-3100, 414, 3000	450, 170	11, 270	370, 290	370, 340	1.1	0.78
Σ [PCPs]	-2700, 250, 1600	-2300, 360, 1000	-1300, 330, 1600	-1500, 55, 1400	230, 120	230, 130	330, 130	42, 190	2.7	0.43
Σ [Phthalates]	-1200, 130, 830	-4700, 120, 870	-2900, 230, 953	-3200, 230, 810	-28, 95	-320, 240	-56, 180	-79, 240	1.9	0.60
Σ [Pesticides]	-940, 120, 580	-3200, 88, 1200	-1200, -11, 2000	-820, 260, 1300	94, 59	-67, 170	-63, 84	290, 120	8.7	0.033
Σ [Flame Retardant]	-100, 22, 280	1.6, 29, 760	-6.2, 26, 290	-79, 20, 360	39, 11	86, 34	49, 14	62, 28	1.2	0.74
Σ [Pharmaceutical]	-6.6, 0, 690	-87, 0, 160	-20, 0, 390	-61, 0, 120	61, 26	31, 13	56, 21	6.3, 10	3.9	0.27
Σ [Industrial]	-40, 30, 270	-91, 38, 370	-90, 42, 260	-7.4, 15, 150	51, 11	46, 16	53, 13	34, 11	3.23	0.37
**Σ [63 PAH method]**	-217, 1.28, 26.3	-16.6, 0.255, 19.7	-15.4, 0.0171, 33.7	-14.3, 3.85, 162	-5.70, 6.48	0.247, 1.25	1.54, 1.66	15.3, 10.2	5.2	0.16
Σ [2-ring PAH]	-214, -0.299, 15.1	-16.2, -0.283, 18.3	-15.9, -1.02, 5.68	-7.77, 0.261, 166	-6.41, 6.34	-0.235, 1.08	-1.36, 0.844	10.9, 10.5	1.85	0.605
Σ [3-ring PAH]	-14.2, 0.898, 10.6	-4.34, 0.115, 4.61	-1.53, 0.312, 41.4	-5.03, 0.243, 41.9	0.408, 0.620	0.110, 0.359	2.46, 1.69	3.44, 2.76	1.13	0.771
Σ [4-ring PAH]	-0.863, 0.203, 1.89	-0.810, 0.0974, 2.98	-0.461, 0.348, 1.59	-2.76, 0.399, 10.9	0.216, 0.0861	0.304, 0.156	0.369, 0.0894	1.11, 0.739	4.40	0.222
Σ [5-ring PAH]	-0.171, 0.0173, 0.347	-0.0466, 0, 0.0337	-0.129, 0, 0.185	-2.31, 0.0342, 0.353	0.0345, 0.0138	0.0328, 0.0170	0.0266, 0.0136	-0.104, 0.151	1.65	0.648
Σ [6&7-ring PAH]	0.00302, 0.0240, 0.530	-0.0123, 0.0196, 0.391	0, 0.0249, 0.263	-0.597, 0.0241, 0.121	0.0535, 0.0174	0.0353, 0.0159	0.0417, 0.0112	-0.0175, 0.0411	2.61	0.456
Σ [parent PAH]	-214, 0.692, 5.55	-16.5, 0.0396, 17.9	-14.2, 0.538, 27.7	-8.70, 0.401, 44.3	-5.65, 6.33	0.270, 1.09	1.38, 1.33	6.87, 3.76	1.24	0.744
Σ [alkylated PAH]	-13.9, -0.104, 20.5	-6.13, -0.0412, 1.64	-5.39, -0.168, 5.92	-5.09, 0.278, 117	-0.183, 0.805	-0.245, 0.324	-0.0923, 0.471	8.02, 7.31	2.56	0.464

*** (*p* < 0.008)**

Table S8. Summary statistics for the difference in the sum concentration of exposures for the 1,500 screening method, 63 PAH method, and their associated chemical categories by participants exposure to home flooding. Participants either did not have flooded homes, had a flooded home and stayed living in that home during flood remediation, or had a home that flooded and moved to an unflooded home. Differences were calculated by subtracting individual estimated baseline concentrations from the paired post-hurricane concentration. A positive value indicates a higher concentration post-hurricane.

	**Min, Median, Max (nmol/WB)**			**Mean (nmole/WB), SE**				
Target Analyte	House Didn't Flood	House Flooded	House Flooded & Moved	House Didn't Flood	House Flooded	House Flooded & Moved	Kruskal Wallis Statistic	P value
**Σ [1,500 screen]**	-4300, 760, 2700	-3100, 170, 2600	-2200, 640, 3000	260, 400	70, 200	530, 160	3.4	0.19
Σ [PCPs]	-640, 360, 1600	-2700, 100, 1600	-1300, 290, 1400	350, 120	49, 140	320, 80	2.6	0.27
Σ [Phthalates]	-4700, 200, 860	-3200, 160, 950	-1200, 130, 830	-300, 330	-220, 150	49,75	0.73	0.69
Σ [Pesticides]	-1500, 120, 1300	-820, 56, 1200	-3200, 57, 570	34, 170	86, 71	30, 84	0.15	0.93
Σ [Flame Retardant]	1.6, 25, 410	-79, 29, 760	-100, 19, 360	83, 29	59, 21	43, 12	1.15	0.56
Σ [Pharmaceutical]	-9.1, 0, 320	-87, 0, 690	-110, 0, 460	41, 20	39, 21	49, 16	0.33	0.85
Σ [Industrial]	-90, 43, 260	-7.4, 25, 370	-91, 32, 270	54, 17	43, 11	49, 8.9	2.2	0.34
**Σ [63 PAH method]**	-304, 0.307, 33.7	-15.7, 0.816, 162	-217, 1.39, 26.3	2.84, 1.94	6.38, 4.58	-4.36, 5.04	0.875	0.646
Σ [2-ring PAH]	-8.00, -0.126, 3.28	-7.77, -0.373, 166	-214, -0.629, 18.3	-0.493, 0.567	4.72, 4.55	-5.67, 4.93	1.17	0.556
Σ [3-ring PAH]	-1.53, 0.276, 41.4	-14.2, 0.0493, 41.9	-5.19, 0.8466, 10.6	3.04, 2.30	1.05, 1.31	0.898, 0.367	2.98	0.226
Σ [4-ring PAH]	-0.186, 0.130, 0.993	-2.76, 0.251, 10.9	-0.838, 0.260, 2.52	0.243, 0.0829	0.616, 0.332	0.329, 0.0920	0.793	0.673
Σ [5-ring PAH]	-0.0466, 0, 0.153	-2.31, 0.0196, 2.65	-0.171, 0.0173, 0.353	0.0183, 0.0117	-0.0285, 0.0652	0.0384, 0.0143	0.168	0.432
Σ [6&7-ring PAH]	0, 0.0209, 0.0663	-0.597, 0.0249, 0.391	-0.0123, 0.0228, 0.530	0.0242, 0.00404	0.0240, 0.0219	0.0479, 0.0137	0.423	0.809
Σ [parent PAH]	-3.83, 0.685, 27.7	-8.70, 0.381, 44.3	-214, 0.468, 17.9	2.11, 1.56	3.36, 1.70	-4.62, 4.93	0.595	0.743
Σ [alkylated PAH]	-1.12, 0.0102, 5.92	-13.9, -0.182, 117	-5.39, -0.0303, 20.5	0.555, 0.456	2.72, 3.22	0.0780, 0.568	0.721	0.697

*** ($p < 0.008$)**

Table S9. Summary statistics for the difference in the sum concentration of exposures for the 1,500 screening method, 63 PAH method, and their associated chemical categories by age group. Age group categories were based off of participant age during the post-hurricane timepoint. Differences were calculated by subtracting individual estimated baseline concentrations from the paired post-hurricane concentration. A positive value indicates a higher concentration post-hurricane.

	**Min, Median, Max (nmol/WB)**			**Mean (nmole/WB), SE**				
Target Analyte	Youth 9-17 yo	Adults 18-64 yo	Elderly >64 yo	Youth 9-17 yo	Adults 18-64 yo	Elderly >64 yo	Kruskal Wallis Statistic	P value
**Σ [1,500 screen]**	-880, 1200, 2700	-4300, 230, 2600	-1200, 860, 3000	1000, 440	38, 170	800, 170	8.5	0.015
Σ [PCPs]	-1000, 470, 1600	-2700, 240, 1600	-450, 393, 1400	370, 300	140, 89	400, 95	1.7	0.42
Σ [Phthalates]	-1700, 390, 860	-4700, 10, 953	-1600, 190, 834	-27, 340	-240, 120	180, 89	4.0	0.13
Σ [Pesticides]	-120, 250, 1300	-3200, 104, 1200	-770, 26, 690	410, 220	-2.2, 75	75, 63	1.1	0.59
Σ [Flame Retardant]	14, 210, 413	-79, 23, 410	-101, 19, 360	200, 51	47, 13	41, 16	10	**0.006****
Σ [Pharmaceutical]	-2.4, 0, 690	-110, 0, 170	-61, 0, 390	33, 25	41, 14	54, 21	0.1	0.95
Σ [Industrial]	23, 42, 120	-91, 27, 370	-40, 41, 160	62, 14	46, 8.9	48, 8.2	3.6	0.17
**Σ [63 PAH method]**	-4.85, -0.391, 5.55	-217, 0.987, 162	-15.7, -0.0535, 22.8	0.309, 1.25	0.717, 4.27	1.76, 1.46	0.398	0.819
Σ [2-ring PAH]	-6.50, -0.434, 5.68	-214, -0.338, 166	-7.77, -0.643, 23.7	-0.608, 1.38	-1.50, 4.17	0.742, 1.27	0.116	0.944
Σ [3-ring PAH]	-0.636, 0.0592, 2.39	-5.19, 0.495, 41.9	-14.2, 0.336, 11.9	0.524, 0.449	1.80, 0.927	0.409, 0.852	0.369	0.831
Σ [4-ring PAH]	-0.0507, 0.354, 0.590	-2.76, 0.208, 10.9	-0.863, 0.211, 3.63	0.329, 0.0859	0.377, 0.180	0.557, 0.202	0.699	0.705
Σ [5-ring PAH]	-0.0466, 0.0381, 0.0796	-2.31, 0.0117, 0.347	-0.251, 0.0175, 0.353	0.0286, 0.0176	0.000636, 0.0369	0.0278, 0.0218	0.298	0.862
Σ [6&7-ring PAH]	0.0200, 0.0253, 0.0740	-0.597, 0.0240, 0.530	0.00313, 0.0184, 0.121	0.0351, 0.00747	0.0378, 0.0152	0.0266, 0.00509	2.21	0.332
Σ [parent PAH]	-1.17, 0.538, 4.38	-214, 0.648, 44.3	-2.39, -0.125, 26.0	0.907, 0.717	-1.43, 3.44	1.81, 1.22	1.59	0.452
Σ [alkylated PAH]	-5.39, -0.0364, 1.00	-6.13, -0.0606, 117	-13.9, -0.0448, 5.30	-0.795, 0.811	1.96, 1.82	-0.387, 0.720	0.480	0.787

*** ($p < 0.008$)**

Table S10. Summary statistics for the difference in the sum concentration of exposures for the 1,500 screening method, 63 PAH method, and their associated chemical categories by gender. Differences were calculated by subtracting individual estimated baseline concentrations from the paired post-hurricane concentration. A positive value indicates a higher concentration post-hurricane.

	**Median (nmol/WB)**		**Mean (nmole/WB), SE**			
Target Analyte	Female	Male	Female	Male	Mann-Whitney U	P value
**Σ [1,500 screen]**	-3100, 410, 2700	-4300, 450, 3000	310, 145	311, 270	1000	0.98
Σ [PCPs]	-2700, 260, 1600	-1300, 300, 1400	190, 87	310, 100	920	0.46
Σ [Phthalates]	-3200, 200, 870	-4700, 70, 950	-59, 91	-250, 210	880	0.31
Σ [Pesticides]	-3200, 75, 1300	-810, 85, 800	28, 74	93, 61	990	0.87
Σ [Flame Retardant]	-100, 27, 760	-0.89, 22, 360	62, 14	44, 14	920	0.45
Σ [Pharmaceutical]	-110, 0, 460	-30, 0, 690	38, 11	57, 27	960	0.68
Σ [Industrial]	-91, 32, 370	-7.0, 38, 160	47, 8.4	49, 8.0	930	0.49
**Σ [63 PAH method]**	-16.6, 0.640, 162	-217, 1.30, 33.7	3.72, 2.47	-5.69, 7.71	975	0.763
Σ [2-ring PAH]	-16.2, -0.338, 166	-214, -0.775, 18.3	2.13, 2.44	-8.02, 7.44	950	0.622
Σ [3-ring PAH]	-5.19, 0.215, 41.9	-14.2, 0.825, 41.4	1.12, 0.683	1.88, 1.55	871	0.272
Σ [4-ring PAH]	-2.76, 0.218, 10.9	-0.863, 0.251, 2.52	0.457, 0.181	0.332, 0.107	990	0.851
Σ [5-ring PAH]	-2.31, 0.00767, 0.337	-0.171, 0.0281, 0.353	-0.00857, 0.0346	0.0539, 0.0194	812	0.119
Σ [6&7-ring PAH]	-0.597, 0.0218, 0.391	0.00555, 0.0249, 0.530	0.0213, 0.016	0.0669, 0.0199	739	0.0336
Σ [parent PAH]	-16.5, 0.505, 44.3	-214, 0.647, 27.7	1.82, 0.956	-5.82, 7.56	1010	0.973
Σ [alkylated PAH]	-6.13, -0.133, 177	-13.9, 0.0942, 5.92	1.64, 1.72	-0.0240, 0.602	869	0.265

*** (p < 0.008)**

Table S11. Summary statistics including results from Wilcoxon matched-pairs signed rank tests corresponding to the chemical categorization utilized in the 1530 screening method. PAHs were excluded due to redundancy with the 63 PAH method, and the categories dioxins/furans and PCBs were excluded due to low detection frequencies.

Chemical Category	Mean Σ [category (nmol/WB)]		Difference (PF-EB)	SE of Difference	Sum of Signed rank (W)	P -value
	Post- Flood	Estimated Baseline				
Personal Care Products	860	640	220	68	2600	<0.0001****
Phthalates	600	710	-110	89	550	0.34
Pesticide	320	270	47	55	1700	0.0034**
Industrial	92	44	48	6.4	4400	<0.0001****
Flame Retardant	81	25	56	11	4500	<0.0001****
Pharmacological	61	17	44	11	1200	<0.0001****
Σ[analytes]	2000	1700	310	130	1700	0.0034**

* ($p < 0.05$), ** ($p < 0.01$), *** ($p < 0.001$), **** ($p < 0.0001$)

Table S12. Summary statistics including results from Wilcoxon matched-pairs signed rank tests for total PAHs detected across timepoints, PAH ring size, and parent/alkylated status.

Ring size	Mean Σ [category (nmol/WB)]		Difference (PF-EB)	SE of Difference	Sum of Signed rank (W)	P -value
	Post- Flood	Estimated Baseline				
Σ[PAH]	11.12	10.15	0.9627	2.865	1180	0.0393*
PAH Ring Size						
2 rings	4.97	5.81	-0.847	2.79	-1020	0.0759
3 rings	4.87	3.52	1.34	0.659	1570	0.00301**
4 rings	1.13	0.712	0.421	0.131	3050	<0.0001****
5 rings	0.08460	0.07485	0.009744	0.02523	1700	<0.0001****
6 & 7 rings	0.06637	0.03173	0.03464	0.01023	4180	<0.0001****
Parent vs. Alkylated						
Parent PAH	6.34	6.53	-0.188	2.32	2030	0.000328***
Alkylated PAH	4.77	3.62	1.15	1.23	-250	0.665

* ($p < 0.05$), ** ($p < 0.01$), *** ($p < 0.001$), **** ($p < 0.0001$)

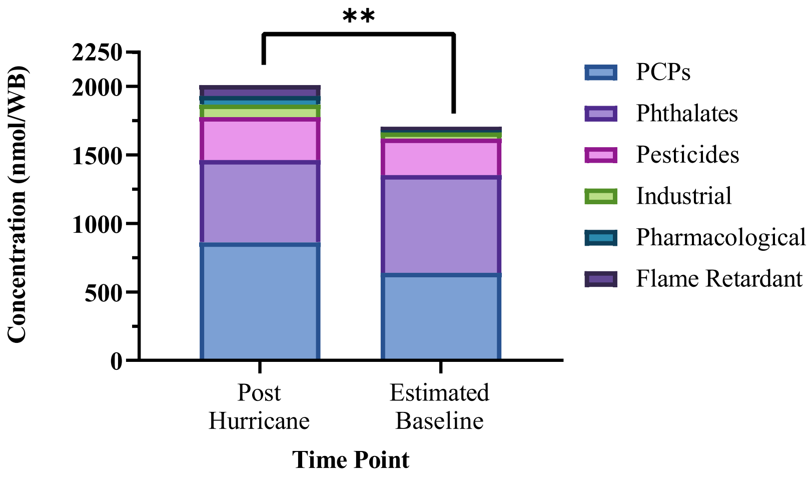

Figure S1. The sum total of chemicals detected within the 1530 screening method post hurricane and at the estimated baseline delineated by chemical classification. * ($p < 0.05$), ** ($p < 0.01$), *** ($p < 0.001$), **** ($p < 0.0001$).

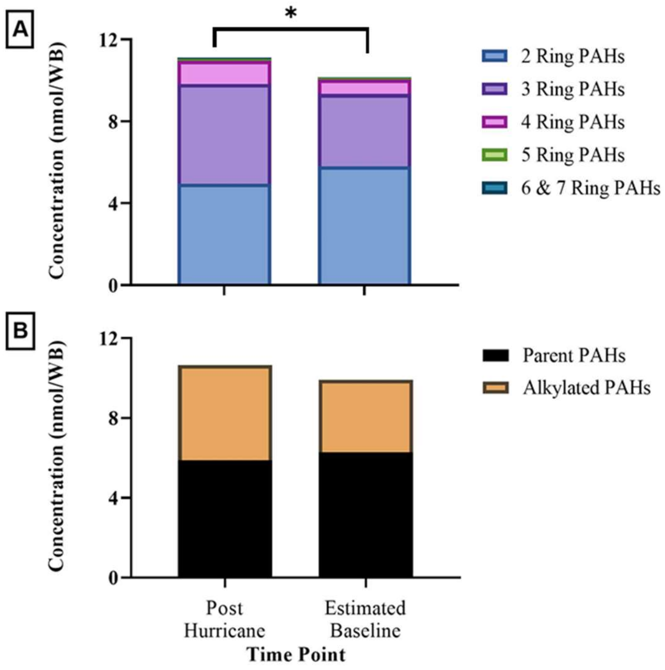

Figure S2. The sum total PAHs detected within the 63 PAH method post hurricane and at the estimated baseline delineated by a) ring size and b) parent versus alkylated status. * ($p < 0.05$), ** ($p < 0.01$), *** ($p < 0.001$), **** ($p < 0.0001$).

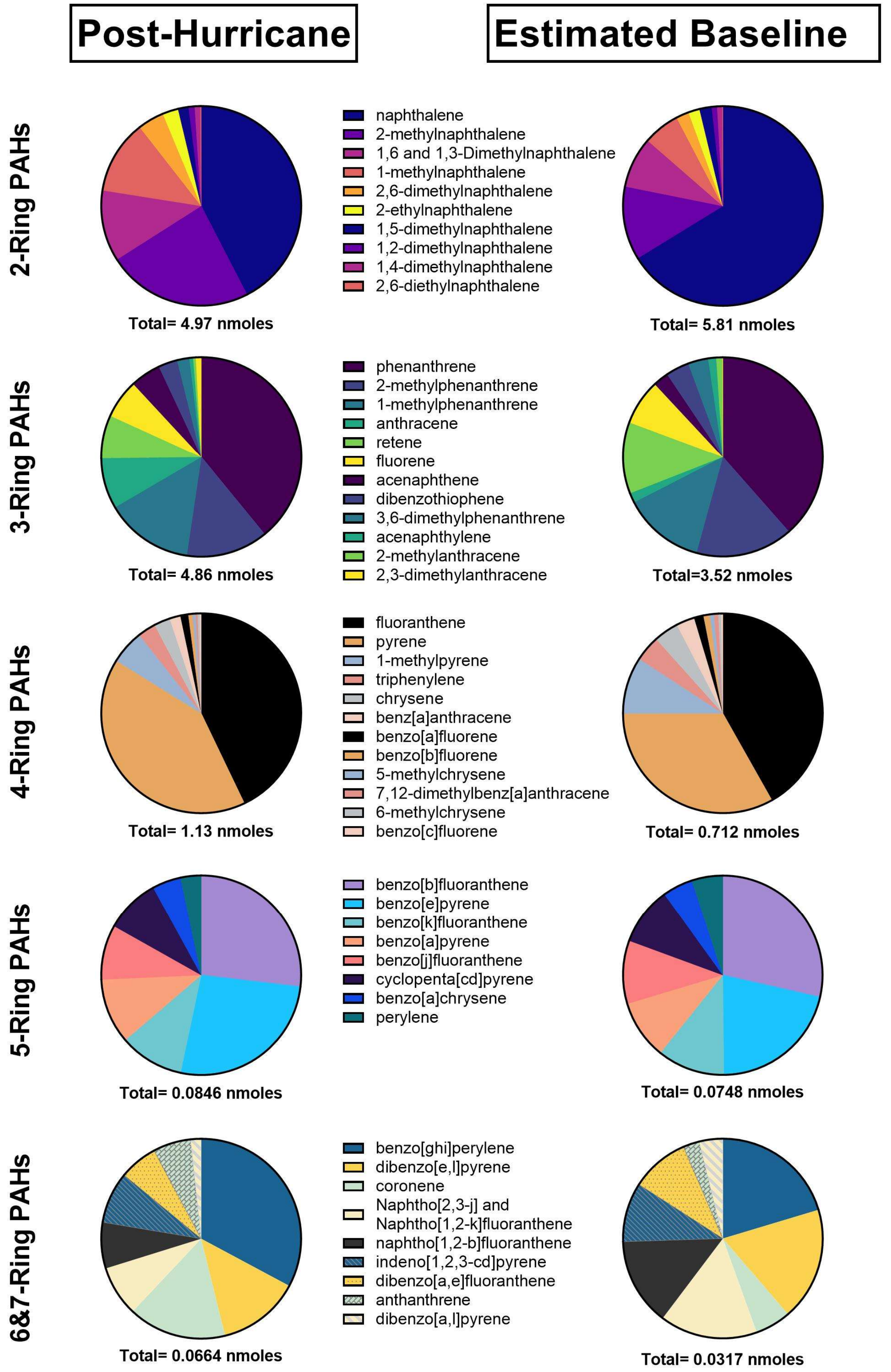

Figure S3. Chemical composition of PAHs across both time points, associated with Figure 4.

Table S13. Comparison of detection frequencies of commonly detected chemicals across Hurricane Harvey timepoints as well as other studies that utilized the same analytic method done within the continental U.S. that had largely adult populations reported in Dixon et al. 2018.

Frequently detected chemicals	% detected in pilot study (n=26)	% detected post hurricane (n=173)	% detected at estimated baseline (n=239)	% detected in other studies with same method (n=59)
**Butyl benzyl phthalate[a]**	100%	96%	98%	76%
**Galoxolide**	100%	95%	98%	97%
**Di-n-butyl phthalate**	85%	93%	100%	93%
**Diethyl phthalate**	92%	91%	77%	95%
**Butylated hydroxytoluene**	92%	91%	85%	95%
**N,n-diethyl-m-toluamide[a]**	81%	86%	92%	64%
**Tonalide**	85%	85%	89%	80%
**Lilial**	96%	85%	88%	76%
**Benzophenone**	92%	84%	84%	90%
**Diisobutyl phthalate**	85%	82%	100%	81%
**Triphenyl phosphate[a]**	88%	80%	76%	49%
**Anthracene[b]**	65%	73%	29%	32%
**Amyl cinnamal**	85%	69%	69%	63%
**1-methylnaphthalene**	31%	67%	30%	15%
**Ethylene brassylate (musk nn)**	39%	66%	85%	84%
**2,4-di-tert-butylphenol[b]**	62%	65%	19%	10%
**Permethrin[a]**	54%	52%	51%	19%
**Di-n-nonyl phthalate**	69%	50%	85%	85%
**B-ionone**	62%	49%	59%	44%
**Benzyl salicylate**	65%	49%	95%	71%

Compounds that have higher frequencies of detection across all Houston timepoints[a], and higher detection frequencies following the hurricane[b].
